# ToxPoint: The indispensable role of zebrafish as a new approach methodology (NAM) in toxicology

**DOI:** 10.1093/toxsci/kfaf121

**Published:** 2025-09-01

**Authors:** Robyn L Tanguay

**Affiliations:** Sinnhuber Aquatic Research Laboratory, Department of Environmental and Molecular Toxicology, Oregon State University, Corvallis, OR 97333, United States

The landmark National Academy of Sciences report, *Toxicity Testing in the 21st Century: A Vision and a Strategy (*NRC 2007*)*, alongside subsequent updates (NRC 2017, 2023) have challenged the toxicology and risk assessment communities to modernize their approaches by integrating the best available science to better protect human health. In response, substantial global investment has accelerated the development of New Approach Methodologies (NAMs) aimed at improving human health protection and, ideally, also reducing or replacing the use of animals in research, drug discovery, and chemical safety testing. Recent policy statements from US agencies—including the FDA, NIH, and EPA—underscore an urgency to mainstream NAMs within regulatory and research frameworks, with the shared goal of modernizing chemical safety assessments and safeguarding public health.

The central aim of risk assessment remains unchanged: To evaluate and predict the likelihood and severity of adverse health effects resulting from chemical exposures. At its core, this discipline seeks to make informed decisions about the safety or hazard of chemicals for whole organisms—ultimately, for humans.

A prevailing criterion for modern NAMs is that they center on human-based science. This is logical considering the well-known genetic, anatomical, physiological, and metabolic differences between humans and other vertebrates. Human cell models, organoids, tissue chips, and computational approaches are thus increasingly prioritized for their species-specific relevance. However, these systems lack the full biological complexity of intact organisms, missing essential multi-organ interactions and systemic responses integral to the manifestation of chemical toxicity. Critical adverse effects, particularly those arising from unexpected or secondary mechanisms, may not be captured by reductionist *in vitro* systems alone. Do we really know that utilizing human genetics is always more important than capturing biological complexity when making chemical use decisions? Chemical exposures in whole animals frequently yield unexpected outcomes, underscoring the need to be watchful for such surprises. While no single NAM can fully predict the effects of chemicals on an entire organism, incorporating whole-organism models alongside other NAMs serves as an important safeguard to detect unforeseen toxicity and elucidate underlying mechanisms.

## Biological and technical advantages of zebrafish

As a result of substantial international investments over the past 25 years, the zebrafish (*Danio rerio*) has cemented its position as a cornerstone of 21st-century biomedical science, exemplifying the promise of NAMs. While public and regulatory attitudes often lag scientific reality, particularly regarding the ethical status of fish, the zebrafish model stands out for its unique combination of biological complexity, relevance, experimental efficiency, and alignment with the 3Rs principles (Replacement, Reduction, Refinement). Early life stage zebrafish represent not merely a supplement, but a transformative alternative to traditional mammalian models. The use of zebrafish in laboratory research has grown exponentially over the past 2 decades. In 1988, there were only 26 academic papers featuring zebrafish; now, this number has surged to over 6,000 annual publications. A Web of Science search for “zebrafish” from all fields revealed 56,816 publications from 2002 to 2023 alone. When restricted to “zebrafish and toxicity,” there were 14,483 publications from 2002 to 2023 indicating the increasing preference for zebrafish as a research model in place of traditional mammals like mice and rats (Zhao et al. 2024).

Many published reviews highlight why researchers have moved to zebrafish for human-relevant biomedical research. Briefly, zebrafish share approximately 70% of their genes with humans, and over 80% of human disease-associated genes have functional zebrafish orthologs (Howe et al. 2013). Genetic homology underpins their translational value in modeling cancers, cardiovascular disorders, neurological diseases, and metabolic syndromes. Zebrafish are not merely a “lesser” vertebrate model, but a uniquely balanced one. Their liver, heart, and brain develop with striking similarity to humans, enabling detection of organ-specific toxicities and neurodevelopmental disruptions that isolated cells and organoids cannot replicate. Cytochrome P450 enzymes, critical for drug metabolism, share 75% sequence similarity with humans, allowing prediction of metabolic activation and detoxification. Transgenic lines faithfully model diseases from melanoma to muscular dystrophy, while permeability to small molecules simplifies toxicity assessment. Rapid external embryonic development enables researchers to observe organogenesis, toxicity, and disease progression in real time, thanks to the embryo’s optical transparency. This noninvasive window eliminates the need for disruptive procedures required in opaque mammalian models. Their small size, high fecundity, and compatibility with high-throughput robotic platforms allow thousands of compounds to be screened weekly at a fraction of the cost and time of rodent studies. Automated imaging, and phenotyping further enable multiparametric analysis of mortality, malformation, behavior, and gene expression, all within an intact vertebrate that captures systemic and developmental effects absent in isolated cells. Data mining of vast zebrafish chemical structure-response databases offers opportunities for AI and systems approaches to understand and predict biological chemical actions.

## Conclusion: Zebrafish embryos as a cornerstone of responsible biomedical research

Efforts to replace animal models with human-centric *in vitro* systems are commendable, but risk prematurely discarding essential tools. Zebrafish embryos bridge the gap between simple cellular models and higher-order mammalian systems, enabling scalable, ethically progressive research with minimal animal welfare concerns. Their continued use in toxicological science is vital for maintaining scientific rigor, enhancing predictive power, and safeguarding human health. To realize the full benefits of zebrafish as a NAM, the research and regulatory communities must clarify their ethical status, ensure appropriate regulatory frameworks, and promote ongoing refinement to further minimize animal suffering. Far from being a mere compromise, the zebrafish embryo represents a paradigm of scientific progress and ethical responsibility in the 21st century.
